# Strength of Flocs Formed by the Complexation of Lysozyme with Leonardite Humic Acid

**DOI:** 10.3390/polym12081770

**Published:** 2020-08-07

**Authors:** Wan Khairunnisa Wan Abdul Khodir, Azizul Hakim, Motoyoshi Kobayashi

**Affiliations:** 1 Graduate School of Life and Environmental Sciences, University of Tsukuba, 1-1-1 Tennoudai, Tsukuba 305-8572, Ibaraki, Japan; s1830239@s.tsukuba.ac.jp; 2 Department of Soil Science, University of Chittagong, Chittagong 4331, Bangladesh; ahakimsoil@cu.ac.bd; 3 Faculty of Life and Environmental Sciences, University of Tsukuba, 1-1-1 Tennoudai, Tsukuba 305-8572, Ibaraki, Japan

**Keywords:** floc breakage, humic substance, protein, aggregation

## Abstract

Aggregation and aggregates properties of natural organic and nanosized macromolecules such as humic substances and proteins are crucial to explore so-called colloid-mediated transport and the fate of substances in soil and water environments. Therefore, the aggregation and dispersion, charging, and floc strength of lysozyme (LSZ)–leonardite humic acid (LHA) flocs were experimentally investigated. The experiments were performed in different salt concentrations and LSZ to LHA mass ratios as a function of pH. We obtained the stronger flocs at pH 4.4, where the isoelectric point (IEP) of the complex with the mass ratio 2.5 was confirmed. Thus, the aggregation of LSZ–LHA flocs is mainly caused by charge neutralization. We obtained the floc strength of 4.7 nN around IEP at low salt concentration of 3 mM, which was stronger than 2.8 nN in high salt concentration of 50 mM. The effect of salt concentration can be rationalized by charge-patch attraction at low salt concentration. With increasing mass ratio, the IEP shifted to higher pH. This is due to the increase in positive charge from LSZ in the mixture. The effect of the LSZ to LHA mass ratio on the maximum strength was weak in the range studied.

## 1. Introduction

In soil and water environments, there are a lot of colloidal and nanosized particles and macromolecules such as clays and natural organic matters. It is of great importance to predict and control the transportation of these particles. The movement of such particles and macromolecules through a soil pore entirely depends on the size of the particles [1]. The size varies due to their aggregation. The aggregation and dispersion are generally discussed on the basis of the Derjaguin–Landau–Verwey–Overbeek (DLVO) forces [2,3], which are composed of van der Waals and electrical double layer (EDL) forces. In addition, hydrodynamic force can disaggregate the floc/aggregate during the transportation. Therefore, the strength of a floc/aggregate against breakage [1,4,5,6,7,8] is considered as an important parameter to predict the movement of these particles in soil and water environments.

Among the charged nanosized macromolecules, proteins have an important role in biogeochemical cycles and indicator of soil quality [9]. However, there are pathogenic types of proteins such as transmissible spongiform encephalopathies (TSEs), pharmaceutical protein, and insecticidal protein toxin (Cry protein from genetically modified crops), which can cause environmental hazard through the soil system [10,11,12,13,14,15,16,17]. These prion proteins have a tendency to bind to the solid particles, minerals, and natural organic matters (NOMs) [12,14,15,16]. This binding affects various behaviors of proteins such as transport and reactivity. The stability, transport, and function of these proteins in soil and water environments can be influenced by the interaction with clay or other colloid fraction in soil especially humic acid [18]. Humic acids are charged macromolecules and one of the hydrophobic fraction of natural organic matter with a heterogeneous characteristic containing carboxylic and phenolic groups, aliphatic, carbohydrates, amides, and aromatic groups [19]. Humic acid plays a role as a carrier in soil and water systems for transportation and distribution of minerals to plants, as well as the contaminants [6] and pathogens. A strong affinity between protein and humic acid, as mentioned above, can lead to the variation in the spread of disease-causing agent and jeopardize the plant and animal life.

While humic acid (HA) has received great attention previously, more works are needed on the relationship between humic acid and protein. Previously, the study on the interaction of lysozyme (LSZ) and HA under the influence of several parameters such as salt concentration, pH, order of addition, and the mass ratio of LSZ to HA has been carried out by using dynamic light scattering (DLS) and isothermal titration calorimetry (ITC) [20]. In the earlier study on the aggregation of humic acid and protein by the Tan et al. [21] group, LSZ, a hydrophilic protein containing hydrophobic parts, an amine group, and both positive and negative charges [22], has been used as a model protein. Further research on the humic acid and protein complex, the Tan group [20] concluded the importance of electrostatic attraction between LSZ and HA, where the largest size aggregates were obtained around isoelectric point at low salt concentration. They also claimed the involvement of hydrophobic interaction between these particles. In this case, unfortunately, the size by dynamic light scattering would be inadequate due to the detection limit [12]. Protein as a complex macromolecular contains an active site to hold an enzyme. By considering this characteristic, Li et al. [23] recognized the effect of aggregation with humic acids on the enzyme activity. The aggregation of protein-humic acid potentially hindered the enzyme activity depending on the properties of the surrounding medium and protein. As an extension to this area of research, a study on the transportation of LSZ–HA aggregates through the quartz sand has clarified that the size of LSZ–HA aggregates is crucial to pass through a medium [12]. For instance, larger aggregates potentially remain in the medium compared to the smaller aggregates. The size of aggregates/flocs is affected by the floc strength against the hydrodynamic rupturing force. At this moment, however, systematic studies on the properties of protein-HA flocs are still lacking.

Recently, Hakim et al. [6,24] published the results on the study of the effect of the pH, tail length of the surfactant, divalent cation, and hydrophobicity of humic acid on the strength of natural organic matter flocs/aggregates. The strongest floc is mainly obtained around the isoelectric point (IEP), where the EDL repulsive force is negligible. They also reported on how the hydrophobicity as additional interaction force contributes the aggregation and aggregate/floc strength. The hydrophobic interaction has a capability to dominate the aggregation behavior of humic acid [6,25,26]. While they investigated the effect of pH, humic substances hydrophobicity and ionic strength on the charging, aggregation, and aggregate strength of humic substances, they have not investigated the effect of mass ratio, ionic strength of salts, and the involvement of proteins on the floc strength. Considering the lack of previous investigation, we have focused on the effect of particles relative concentration or the mass ratio and ionic strength.

Transport of the particles and aggregates entirely depends on their size. During the transportation, the breakup of aggregate/floc probably occurs by hydrodynamic force, when it is larger than the strength force of the flocs [4,27,28]. The flocs with strong floc strength would be difficult to migrate through the porous system of soil, meanwhile the flocs with weak strength is easier to move through the soil pore. This could also affect the sedimentation of the flocs in flow fields. Previous studies on the physical analysis on the floc strength have been reported [7,28], however, numerical values of floc strength of protein-HA floc has not yet been published. Therefore, this paper reveals the novel numerical values of floc strength of protein-NOM flocs from the breakage experiment of LSZ–LHA flocs for the first time. Since the surrounding condition of the aggregates can affect the aggregation, we focus on the effect of pH, KCl concentration, and mass ratio on the floc strength of this complex.

## 2. Materials and Methods

### 2.1. Leonardite Humic Acid and Lysozyme

Humic acid used throughout the present experiment was leonardite humic acid (LHA, IS104H, Bowman County, ND, USA) obtained from the International Humic Substance Society (IHSS, Colo., St. Paul, MN, USA). To reduce moisture, the LHA powder was oven-dried overnight at 65 °C. The LHA stock solution (0.25 wt %) was prepared by dissolving the LHA dried powder with KOH solution (Wako 1st grade, Wako Pure Chemical Industries, FUJIFILM Corporation, Osaka, Japan) until fully dissolved. According to IHSS, LHA has 4.76 mmol/g of COOH groups, and in order to fully dissolve LHA, the amount of KOH should be equivalent or more than the number of carboxylic groups of humic acid [29]. The diluted solution using deionized water (Elix, Milipore, Merck KGaA, Darmstadt, Germany) was prepared as secondary stock solution (0.05 wt %) and kept at 4 °C. The concentration of 10 mg/L of LHA (lysozyme-LHA mixture) was maintained throughout all the experiments.

A powder-form lysozyme (LSZ) protein (Sigma Aldrich, L6876-10G, Merck KGaA, Darmstadt, Germany) was used in the experiment as a model protein due to its high structural stability. The lysozyme is an ellipsoidal shape protein with a size between 3.3 and 4.5 nm and has a molecular weight of 14.6 kDa with an isoelectric point around pH 10–11.1 [21,30,31,32,33]. The lysozyme powder was dissolved in deionized water as the stock solution and stored in 4 °C. The solution was prepared freshly every two weeks. The lysozyme solution was directly used without further purification.

An inorganic salt KCl (JIS special grade, Wako Pure Chemical Industries, FUJIFILM Corporation, Osaka, Japan) was used to investigate the effect of salt concentration on floc strength. The solution was filtered (DISMIC 25HP 0.2 µm, ADVANTEC, Toyo Roshi Kaisha Ltd., Tokyo, Japan) after the dilution. The KCl concentrations adopted in the following experiment were from 3 to 50 mM. Another important parameter to observe the floc strength is pH. Therefore, HCl (volumetric analysis grade, Wako Pure Chemical Industries, FUJIFILM Corporation, Osaka, Japan) or KOH were added to control the pH. To avoid CO_2_ contamination, all the solutions were degassed under reduced pressure using vacuum pump (GCD-051X, ULVAC, Kanagawa, Japan) before the experiment.

### 2.2. Electrophoretic Mobility

The electrophoretic mobilities of LSZ–LHA complexes in different KCl concentrations (3 mM, 10 mM, and 50 mM) were measured using Malvern Zetasizer Nano ZS apparatus (Malvern Instrument Ltd., Worcestershire, UK) as a function of pH. The pH of the mixture was adjusted manually with HCl or KOH to obtain the desired pH. The LHA was added as the last step, since the order of addition also influenced the aggregation behavior (Tan et al. [34]) and was sonicated beforehand for 15 min in the cold condition to prevent aggregation amongst LHAs before the interaction with LSZ. All the solutions employed in the experiment have been degassed before use. The bottle was inverted slowly to mix the sample and to initiate the interaction between HAs and LSZs. The mixture was allowed to stand for 24 h before analysis. The mixture was then mixed thoroughly before injection into the cuvette. The experiment was performed at 20 °C. The pH was checked using a pH meter (ELP-035, TOA DKK, DKK-TOA Corporation, Tokyo, Japan). The mass ratio of the mixture LSZ–LHA was written as *C*_LSZ_*/C*_LHA_, where *C* represents the mass concentration of the sample. The concentration of LHA was fixed to 10 mg/L throughout the experiment and the concentration of LSZ was adjusted to obtain mass ratio *C*_LSZ_*/C*_LHA_ = 2.5 and 5. Meanwhile, the concentrations of LSZ at mass ratio 2.5 and 5 are 25 mg/L and 50 mg/L, respectively. A similar procedure was used for the floc breakage experiment under a different *C*_LSZ_*/C*_LHA_ mass ratio as a function of pH. The experiment was performed for independently prepared samples in triplicate.

### 2.3. Observation of Aggregation and Dispersion

Observation on the aggregation–dispersion and formed flocs of LSZ–LHA complex at different KCl concentrations (3 mM, 10 mM, and 50 mM) was performed macroscopically and microscopically as a function of pH. The LSZ–LHA mixtures with the total 5 mL volume at the mass ratio of 2.5 within the range of pH 3–8 were prepared in clear polystyrene bottles. The pH was adjusted with HCl and KOH to reach the needed pH. The mixtures were inverted once to ensure the mixing and the bottles were allowed to stand for 24 h. For the macroscopic observation, a camera (Olympus PEN Lite, E-PL 7, Olympus Corporation, Tokyo, Japan) was placed in front of the series of bottles (3 mM KCl at pH 3–8) and photographed for every 30 min. The objective of macroscopic observation was to check the aggregation–sedimentation state at different KCl concentrations and pH. Microscopic observation was performed after 24 h standing. The mixture was swirled softly to resuspend the sediment before observed under the microscope (Shimadzu BA210E, Moticam 580INT, Shimadzu Corporation, Kyoto, Japan). This observation was purposely to obtain the size of individual aggregates and the aggregate arrangement in every condition.

### 2.4. Floc Breakage and Strength

**Figure 1:**
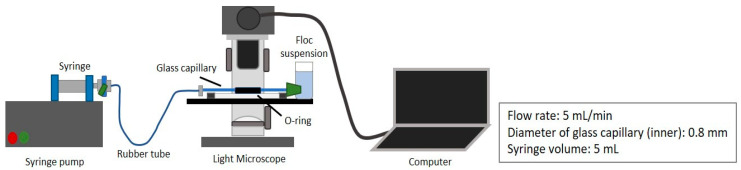
Schematic illustration of floc breakage experiment.

The strength of a floc was measured through the floc breakage by a converging flow to a capillary [6,24] as illustrated in Figure 1.

The bottle containing settled flocs/aggregates in the sample was slowly swirled before being placed into the sample cuvette connected to a tube with minimum damages. A 0.8 mm inner diameter of glass capillary and silicone stopper were installed on the cuvette to create converging flow. Prior to flocs breakage, the syringe pump (Fusion 200, Chemyx, Chemyx Inc., Stafford, TX, USA) was set with a flow rate of 5 mL/min. A microscope was used to observe the broken flocs in the capillary, which was placed in water in an O-ring on a slide glass covered by a cover glass. To reduce the contact between flocs after the breakage, the flow valve was closed while the images were taken. Noted only an image of a single and largest aggregate was taken for further analysis. The selected image was analyzed using ImageJ 1.52a (Java 1.8.0 ver, National Institute of Health, Bethesda, MD, USA), where the maximum and minimum diameter (*d*_maj_ and *d*_min_) of flocs were obtained from a best-fit ellipse to a floc. All the experiments were performed in triplicate.

The evaluation of floc strength against breakage was described by Kobayashi [1,5]. The breakup of a floc probably occurs if the hydrodynamic rupture force on the floc is larger than the strength force of the floc.

*F*_hyd_ ≥ *F*_floc_ (1)

when flocs are subjected to a converging flow to a capillary, floc breakage occurs due to a high elongation rate near the tube entrance during the converging flow to a tube. Kobayashi considered that the highest elongation rate along the centerline *A*_c,max_ to a capillary determines the maximum floc size. The elongation rate *A*_c,max_ is given by

(2) $$A_{c,\max}=\frac{3\sqrt{3}Q}{32\pi R^{3}}$$

where *Q* is volumetric flow rate and *R* is the capillary radius. The converging flow along the centerline is assumed as an axisymmetric straining flow. Blaser [35] showed the hydrodynamic rupturing force with viscosity *µ* acting on an ellipsoidal floc with a surface area *S* in an axisymmetric straining flow at a elongation rate *A* as

(3) $$F_{\mathrm{hyd}}=\frac{C_{\mathrm{hyd}}S\mu A}{2}$$

where *C*_hyd_ is determined by the shape of the ellipsoids.

Consequently, the strength of the flocs is determined from the maximum size of broken flocs. The surface area of the floc as an ellipsoid *S* is calculated by the major and minor lengths (*d*_maj_ and *d*_min_) of the floc, which resemble the maximum surface area of the best-fit ellipsoids *S*_max_ as described in the equation below [36]

(4) $$S=2\pi \left(\frac{ac^{2}}{\sqrt{c^{2}-a^{2}}}\;\mathrm{arc}\cos\frac{a}{c}\right)$$

where 2*a* = *d*_min_ and 2*c* = *d*_maj_. Kobayashi [1] has listed *C*_hyd_, which can be used to calculate (*C*_hyd_*S*)_max_, which is the maximum values of *C*_hyd_*S* of flocs broken by the flow with *A*_c,max_. Following all the consideration, Kobayashi [1] concluded the equation for floc strength as below;

(5) $$F_{\mathrm{floc}}={\left(C_{\mathrm{hyd}}S\right)}_{\max}µA_{c,\max}/2$$

All the strength values in this investigation were evaluated using the equations described above.

## 3. Results and Discussion

### 3.1. Electrophoretic Mobility of the LSZ–LHA Complex

#### 3.1.1. Effect of the KCl Concentration

**Figure 2:**
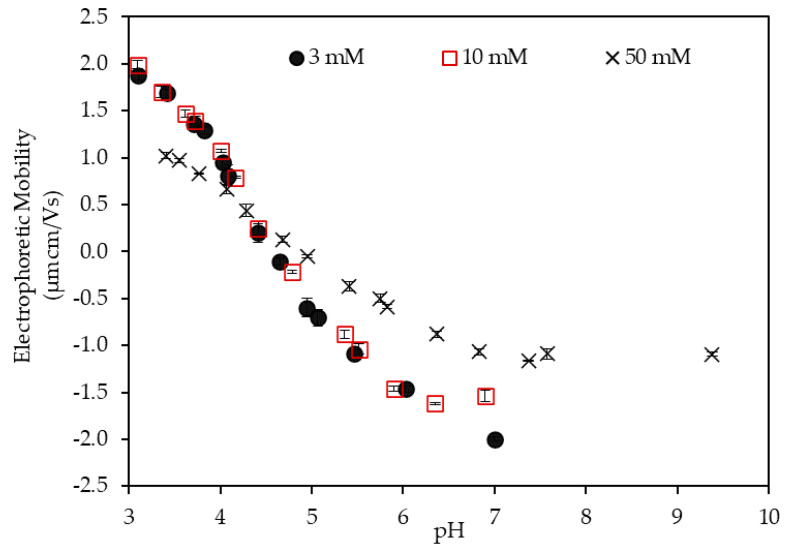
Electrophoretic mobility of the LSZ–LHA complex with mass ratio 2.5 at different KCl concentrations (3 mM, 10 mM, and 50 mM) against pH.

The electrophoretic mobility (EPM) was used to investigate the charging behavior of the LSZ–LHA complex. The measured mobility of the LSZ–LHA complex with a mass ratio at *C*_LSZ_*/C*_LHA_ *=* 2.5 is presented in Figure 2 as a function of pH at different KCl concentrations. The figure shows the obvious charge reversal of the LSZ–LHA suspensions for all the KCl concentrations (3 mM, 10 mM, and 50 mM). The charge reversal of the LSZ–LHA complex from positive to negative mobility for all KCl concentrations is found with increasing pH (Figure 2). At lower pH, the EPM of the complexes at all KCl concentrations appeared as a positive value until reaching zero mobility or IEP at pH 4.5. As the pH gets higher, the mobility becomes negative. In addition, Figure 2 shows the effect of salt on the EPM of the LSZ–LHA complex. This effect was obviously based on the magnitude of EPM data for all KCl concentrations (3 mM, 10 mM, and 50 mM). The EPM at 50 mM KCl was smaller in magnitude compared to the lower KCl concentrations.

The charge reversal of the LSZ–LHA complex was due to the binding of negatively charged LHA to positively charged LSZ at pH 4.5. At lower pH, LSZ had more positive net charges and LHA had fewer negative charges. As a result, the LSZ–LHA complex shows positive EPM at low pH. As the pH gets higher, the LSZ slowly dissociates and the deprotonation occurs, leading to fewer positive charges [21]. This causes LHA to get a negative charge higher than the positive charge of LSZ at higher pH. The surface charge density on the LSZ and LHA at different KCl concentrations as a function of pH is described in Figure S1.

Another reason for charge reversal is the hydrophobic interaction between LSZ and LHA. A comparison study between several types of humic substances in KCl solution [29] has discovered no charge reversal occurs and the mobility of LHA remains negative. Clearly, in this work, the presence of LSZ in the LHA system has established a hydrophobic interaction. The interaction becoming strong around pH 4.5 where the surface charge is the weakest due to charge neutralization, as being pointed out by Sugimoto et al. [37].

The difference in the magnitude of EPM in Figure 2 is noteworthy on the effect of KCl concentration. In Figure S1, the surface charge density of LSZ increased with the increase of salt concentration by the result on the proton binding isotherm of LSZ and LHA. However, in this LSZ–LHA complex system, the low magnitude of EPM at 50 mM KCl is rationalized by the compression of EDL. The addition of high KCl concentration (by 50 mM in this work) caused the production of excess protonated charges but reduced the surface potential, therefore the magnitude of mobility. This occurrence is explained by the Debye length for each KCl concentrations (Table S1). The Debye length was used to estimate the thickness of EDL and was 5.50 nm, 3.05 nm, and 1.36 nm for 3 mM, 10 mM, and 50 mM KCl, respectively. The higher the salt concentration is, the greater the screening effect of the surface potential is. The positive and negative EPM at low and high pH explained the charging trend for 50 mM KCl. Unlike the EPM of 50 mM KCl, we discovered the weak dependent on KCl concentration in the mobility of 3 mM and 10 mM KCl concentrations. Similar results can be found by previous studies [31,38]. At low pH, the positive charge of LSZ is high to cause repulsion but it is reduced by the presence of LHA in the system. For the homogenous colloid system, the effect of salt concentration is more noticeable.

#### 3.1.2. Effect of the Mass Ratio

**Figure 3:**
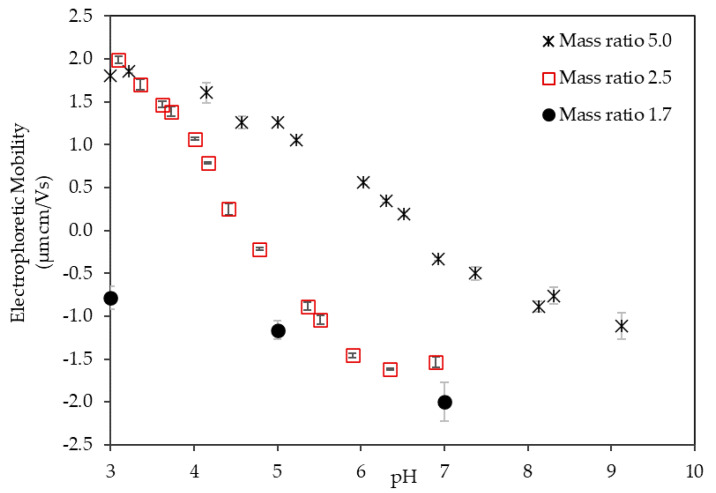
Electrophoretic mobility of the LSZ–LHA complex as a function of pH in 10 mM KCl at different mass ratios (1.7, 2.5, and 5).

The effect of the mass ratio on the EPM of the LSZ–LHA complex in 10 mM KCl concentration is shown in Figure 3. The mass ratio of LSZ to LHA was used in the experiment, where the LHA concentration was fixed to 10 mg/L. The increase in the mass ratio was equivalent to the increase in lysozyme concentration. Figure 3 shows EPM measured at different mass ratios (mass ratio 2.5 and 5), demonstrating a similar charge reversal from positive to negative over pH as in Figure 2. The EPM at the mass ratio 1.7 was negative, but there was an increase in mobility magnitude with pH. We see a visible change in EPM of the LSZ–LHA complex in Figure 3 for all the mass ratios by the change in lysozyme concentration. The EPM at the mass ratio 5, initially, had a small raise at pH 3 before gradually decreasing in mobility as pH increased, meanwhile the mobility at mass ratio 2.5 decreased drastically. With increasing lysozyme concentration (mass ratio 5 > 2.5), the mobility reached IEP at a certain pH. The IEPs were around pH 7.0 and 4.5 at the mass ratio 5 and 2.5, respectively. There was no IEP for mass ratio 1.7 for the selected pH range (pH 3–9). A similar experimental outcome was obtained for LSZ-purified Aldrich humic acid at 5 mM KCl [21]. The proton binding isotherm for different mass ratios (*f* = 0.1 and 0.4) resulted in pH_IEP_ at pH 7.8 and pH 4.2. The result on the effect of mass ratio points out the importance of particle concentration ratio on the charging and thus aggregation behaviors under the influence of pH.

The result from Figure 3 shows a pronounced effect of the mass ratio of LSZ and LHA on EPM behavior, which indicates the importance of LSZ and LHA concentration. For mass ratio 2.5, the ratio of LSZ to LHA was adequately enough for the affinity between opposite charges to neutralize each other. Unlike the mass ratio 2.5, the total net charge for mass ratio 1.7 is negative, which shows the abundant amount of LHA than LSZ in the suspension. Mass ratio 5, on the other hand, with an abundant amount of LSZ than LHA could cause a screening mechanism in the earlier pH.

As mentioned previously, the hydrophobic interaction does take part in the LSZ–LHA aggregation system. The charge reversal occurs at pH 7 and 4.5 for mass ratio 5 and 2.5. This clear distinction on the effect of mass ratio (Figure 3) emphasized the factor of surface charge on charge reversal. A high mass ratio (mass ratio 5) had a higher positive surface charge than mass ratio 2.5, therefore reaching charge reversal at the later pH.

### 3.2. Observation on the Aggregation Behavior of the LSZ–LHA Complex

#### 3.2.1. Effect of KCl Concentration

**Figure 4:**
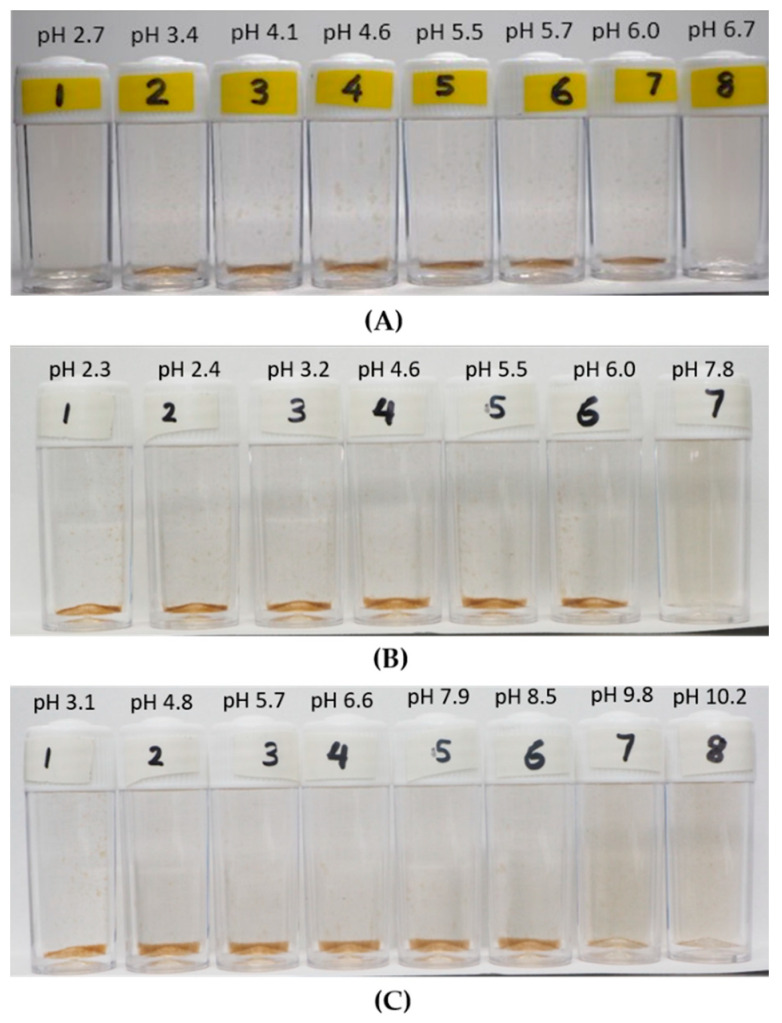
Aggregation of LSZ–LHA complexes on mass ratio *C*_LSZ_/*C*_LHA_ = 2.5 at 3 mM (**A**), 10 mM (**B**), and 50 mM KCl (**C**) concentration under various pHs. The time frame for all images is at 24 h.

The macroscopic observation on the effect of KCl concentration on aggregation–dispersion is displayed in Figure 4, where the photos of the suspension for the LSZ–LHA mixture at mass ratio 2.5 with all KCl concentrations (3 mM, 10 mM, and 50 mM) are shown. This observation shows that the aggregation and sediment formation occurred at different pH depending on the concentration of KCl. The observation on 3 mM KCl concentration exhibited the sedimentation range that appeared from pH 3.4 until 6.0, meanwhile a wider pH range of sedimentation for 50 mM KCl until pH 10. The observation on the aggregation–dispersion behavior is crucial to mark the pH range of aggregation. The early stage of aggregation for all KCl concentrations is shown in Figure S2. The flocs are noticeable after 2 h around pH 4.5, where the condition is near the IEP region, suggesing that the aggregation is quite fast. At this point, the sedimentation is clearly seen and, at the same time, flocs slowly emerged towards the lower and higher pH.

From Figure 4A (taken within 24 h), we can see clear sedimention formation of LSZ–LHA mixtures (bottles 2–7). As the pH goes further away from the IEP range, the sediment is gradually leveling off or thinner. For the 10 mM and 50 mM KCl concentration shown in Figure 4B,C, the sedimentation occurred even below pH 3, which is partly due to self-aggregation of humic acid at low pH. This self-aggregation of humic acid is known to be the effect of hydrophobicity and the hydrogen bond in the interaction between humic acid particles. In addition, protein too contains hydrophobic moiety, which could lead to further aggregation at lower pH. The overall hydrophobicity of lysozyme has been reported by Norde et al. [33], which could affect the adsorption of protein.

The images of individual flocs are displayed in Figure S3 for seeing any differences in the arrangement or structure of the aggregates before the breakage for all KCl concentrations. These flocs displayed in Figure S3 were formed at the mass ratio 2.5 at different KCl concentrations (3 mM, 10 mM, and 50 mM), and the images were captured within the aggregation pH range. The aggregation between humic acid and protein is well endorsed by [39] through spectroscopy analysis (Fourier transform infrared (FT-IR) and nuclear magnetic resonance (NMR)), which reported the formation of the amide bond.

The aggregation–dispersion range found from the macroscopic view in Figure 4 is expectedly in line with EPM data (Figure 2) since the EPM portrays the total surface charge. At the point around IEP pH (around pH 4.5), the sediments are thicker due to higher interparticle attraction between LSZ–LHA particles and causes the formation of open-structured fractal-like flocs and sediment. As the LSZ–LHA suspension moves out of the IEP region, the attraction between LSZ–LHA particles is lesser and thus the aggregation as well as the sedimentation are reduced. This weak interaction results in the formation of thinner sediment near the dispersion range as seen in Figure 4.

#### 3.2.2. Effect of the Mass Ratio

**Figure 5:**
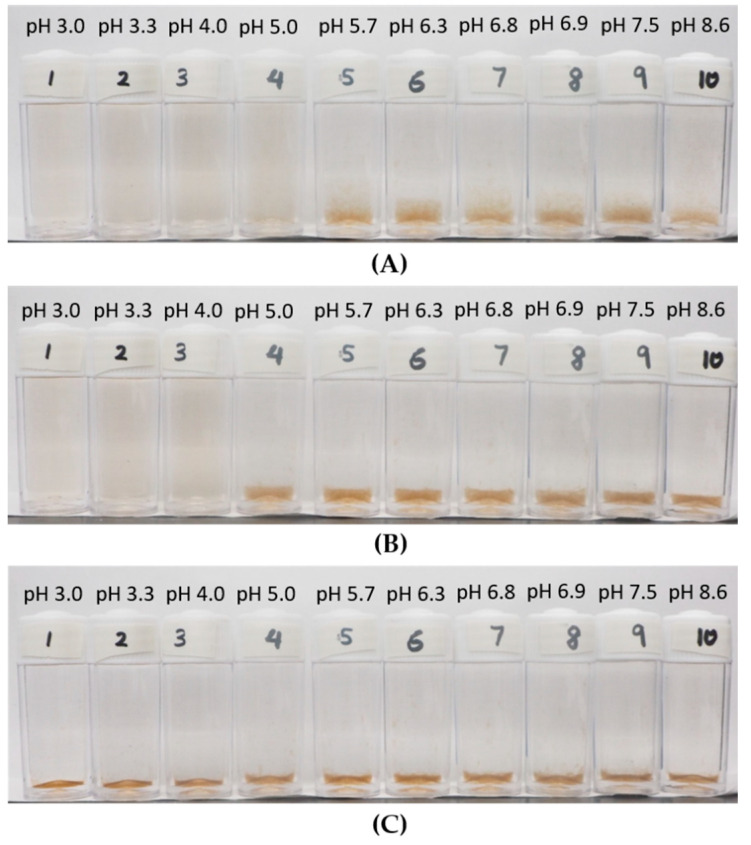
Macroscopic view for the LSZ–LHA complex at mass ratio *C*_LSZ_/*C*_LHA_ = 5 at 10 mM KCl concentration over pH. The time frame images are after 2 h (**A**), after 3 h (**B**) and after 24 h (**C**).

The observation of the LSZ–LHA complexes at different mass ratios was only carried out at the mass ratio 2.5 and 5. The macroscopic observation of the LSZ–LHA complex at mass ratio 5 at 10 mM KCl concentration is displayed in Figure 5. The aggregation–sedimentation started from bottle 5 (pH 5.7) towards the higher pH within 1–2 h. After three hours, obvious aggregated sediments started to appear at bottle 4 (pH 5) and slowly the process leaned toward the lower pH. Fast aggregation in bottles 5–10 points out the charge neutralization effect since the pHs were near the IEP range (Figure 3). Surprisingly, the bottles 1–4 show the formation of aggregates far from the IEP range, which specifies the contribution of hydrophobicity of the humic acid and protein. On the other hand, the aggregation–dispersion behavior for the LSZ–LHA complex at mass ratio 2.5 in 10 mM KCl concentration is different from the suspension at mass ratio 5.

The results of microscopic observation of the flocs at different pH (pH 3.3, 5.0, and 6.8) are shown in Figure S4. The flocs result in different sizes from pH 3 to pH 6.8. The largest floc around 50 μm is detected at high pH (Figure S4C), followed by Figure S4A,B with the flocs around 30 μm and 10 μm, approximately. Furthermore, only the flocs in Figure S4C were aligned with the mobility data, which occurred at zero IEP range. The differences mark the importance of pH, however, the disagreement with mobility data (Figure 3) suggests that different types of interactions were involved other than charge neutralization.

The LSZ–LHA complexes at the mass ratio 2.5 (Figure 4) produced the pronounced flocs around IEP, indicating that the dominant aggregation mechanism is the charge neutralization where the opposite charges counterbalance each other. For the mass ratio 5 (Figure 5), the latter floc formation at pH below 4 indicates the dominance of van der Waals and hydrophobic attraction, where the electrostatic double layer forces became relatively weak. This condition took place by a high concentration of bare LSZ particles. The screening of the LSZ positive charge also featured the mobility data in Figure 3. Szilagyi et al. [40] reported that a high concentration of protein will cause a screening effect despite the number of valence or salt concentration.

At the mass ratio 5, the formation of flocs below the IEP does indicate other interactions such as hydrophobicity and van der Waals effects. This would be because both humic acid and protein contain hydrophobic moiety. Previously, the aggregation of humic acid at low pH has been studied to have a strong hydrophobicity effect. Besides, the homoaggregation by humic acid could take place at low pH as well.

### 3.3. Floc Strength of the LSZ–LHA Complex

#### 3.3.1. Effect of KCl Concentration

**Figure 6:**
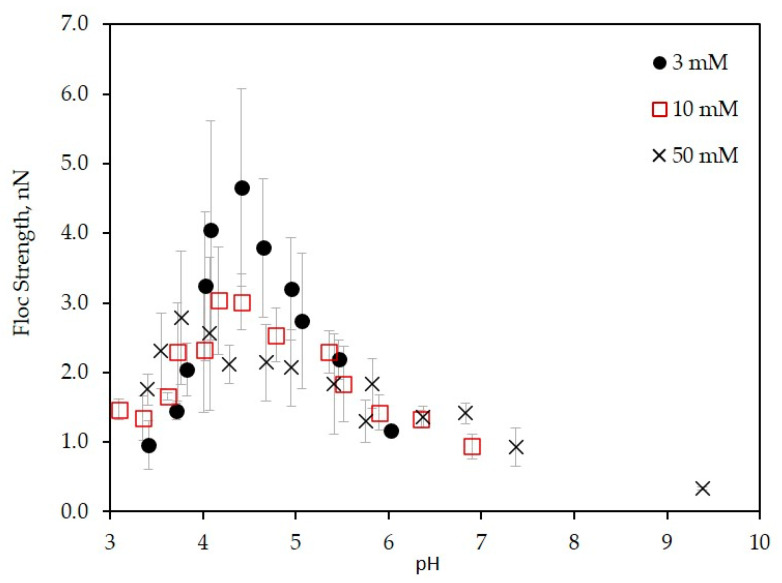
Floc strength of mass ratio CLSZ/CLHA = 2.5 at different KCl concentrations (3 mM, 10 mM, and 50 mM) as a function of pH.

In Figure 6, the floc strengths are plotted as a function of pH at different KCl concentrations. The result shows that the floc strength of the LSZ–LHA complex was strongly controlled by KCl concentration and pH. The strength’s peaks appeared around pH 4.5. The important finding from Figure 6 is that the highest floc strength up to 4.7 nN was obtained at 3 mM KCl followed at 10 mM and 50 mM KCl with a strength around 3.0 nN and 2.8 nN, correspondingly. These peaks arose around the IEP range (pH 4–4.5, except for 50 mM data at pH 3.8) as mentioned in Figure 2. This maximum strength being consistent with the EPM data (Figure 2) justified that the leading mechanism of aggregation was the charge neutralization. The curves of the floc strenght vs. pH were narrow for 3 mM KCl and wider for 50 mM KCl. These trends could be explained by the DLVO theory; the interparticle repulsion is reduced for weakly charged particles at higher salt concentration. We observe that the flocs with stronger strength were formed for lower KCl concentration (3 mM) around the IEP. This was not mere charge neutralization predicted by the classical DLVO theory, within which the strength dominated by the van der Waals force should be constant without electrostatic repulsion at IEP. The error bars presented in Figure 6 were rather large especially around the peak of graph. This is probably because of the presence of the distribution of floc/aggregates strength due to the heterogeneity of LHA and restructuring of flocs and was also because of the probabilistic nature of breakage and floc orientation.

Several studies on the aggregation of colloidal matters induced by the adsorption of oppositely charged substances showed that the aggregation rate or aggregate size near IEP increases with decreasing salt concentration [20,31,41,42,43,44]. Such a tendency agrees with the present data demonstrating the increase of floc strength around IEP with decreasing KCl concentration, meaning the existence of an additional attractive force at low salt concentration. This enhancement of additional attractive non-DLVO forces has been identified as charge-patch attraction [11,13,31,45,46,47,48]. At the IEP range, the oppositely charged particles heterogeneously attach to each other. The attractive double layer forces between oppositely charged local parts in an averagely neutralized floc contribute the formation of stronger flocs. This interaction usually appears at low salt concentration where the attractive double layer is significant [49,50,51]. The charge-patch and double layer attractive forces at low salt concentration are screened by the higher salt concentration. Therefore, we consider that the floc strength becomes weak at higher salt concentration.

Comparing Figure 2 and Figure 6, the peak of the floc strength for each KCl concentrations shifted below the IEP as the KCl concentration getting higher (3 mM, 10 mM, and 50 mM) with pH 4.4, pH 4.2, and pH 3.8, accordingly. This unalignment was less at low KCl concentration since the bonding induced by the charge patch was stronger. The effect of the charge patch did not work at a higher KCl concentration due to the presence of high screening of electrostatic forces. Therefore, the peak of floc strength at 50 mM shifts a bit left to the lower pH from the IEP. Probably, other interactions such as hydrophobic attraction and/or hydrogen bonding become relatively dominant at a lower pH condition at a higher salt concentration.

The study on the effect of KCl concentration on floc strength has suggested the importance of additional non-DLVO forces for the aggregation in the mixture of oppositely charged proteins and humic substance.

#### 3.3.2. Effect of the Mass Ratio

**Figure 7:**
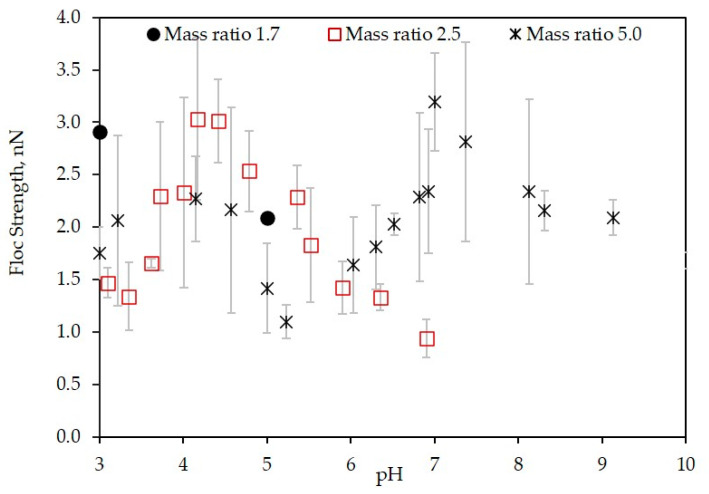
Floc strength of the LSZ–LHA complex at a different mass ratio (1.7, 2.5, and 5) in 10 mM KCl concentration as a function of pH.

The effect of the mass ratio of LSZ–LHA on the strength of flocs was measured at pH from 3 to 10. Figure 7 presents the floc strength obtained at mass ratio *C*_LSZ_*/C*_LHA_ = 1.7, 2.5, and 5 at 10 mM KCl.

The impact of the mass ratio on the floc strength is shown in Figure 7. Foremost, the highest strength was up to 3 nN, which was the maximum strength for LSZ–LHA aggregates at 10 mM KCl despite of any possible interactions involved. The data with mass ratio 5 present a different pattern of floc strength from pH 3-9. That is, the floc strength at mass ratio 5 shows a recurrence formation of strong flocs at both low and high pH, with the highest strength around 2.3 nN and 3.2 nN. The highest strength around pH 7 was comparable with the highest strength at the mass ratio 2.5. Comparing the EPM (Figure 3) and floc strength (Figure 7), the highest strength was initiated by charge neutralization, because the occurrence is near the IEP range.

Contrarily, another peak of floc strength at low pH (around pH 4) was not induced by charge neutralization. The possible interaction that promotes the aggregation is chemical interactions, namely, the hydrophobic interaction and/or hydrogen bonding. Nonetheless, the real mechanisms that cause the aggregation at this point are not clear. The mobility (Figure 3) at this point shows a positive, which normally indicates the existence of EDL repulsion. The presence of distinct aggregation at low pH for mass ratio 5 reveals additional attractive interactions that overcome this repulsion. It appears that the additional intermolecular interactions were influenced by pH through the protonation and deprotonation of carboxylic and amino groups. Moreover, the functional group, which also contains a non-polar amino acid, could initiate a similar hydrophobic interaction.

In the case of mass ratio 5, the multiple peaks of strength emphasized the floc strength induced by charge neutralization was stronger than the floc strength induced by the chemical interaction with electrostatic repulsion. The floc at mass ratio 1.7 shows the highest strength up to 2.9 nN. In the case of mass ratio 1.7, no means of charge neutralization was involved. In the low pH area, lower electrophoretic mobility (Figure 3) indicates the reduction of EDL repulsion. In addition, the aggregation was thoroughly induced by chemical interactions such as van der Waals, hydrogen bonding, hydrophobic interactions and by partial charge-patch attraction overcoming electrostatic repulsion.

## 4. Conclusions

This study broadened the previous research and provided a new approach of evaluating the interaction between protein and humic acid through the quantitative assessment of the strength of flocs of lysozyme (LSZ) and leonardite humic acid (LHA) against breakage by converging flow. The strength of the flocs was investigated as a function of salt concentrations, mass ratio, and pH. We confirmed higher strength around the isoelectric point (IEP) due to charge neutralization removing the double layer repulsion as a main aggregation mechanism. The involvement of the additional non-DLVO force called as the charge patch was discussed. This heterogeneous patch force was only applicable at low salt concentration where the screening of the double layer is the lowest. Through this force, the highest strength of flocs was obtained at 4.7 nN compared to without the additional forces, which reached to 3.2 nN only. Additionally, other forces such as hydrophobic and hydrogen bonding also influence the strength of flocs under an appropriate portion of mass ratio, which gives the strength up to 2.9 nN. This research marks the importance of the intermolecular interaction in the aggregation–dispersion system.

This research indeed provided an appealing insight on the evaluation of flocs strength of protein-humic acid flocs. Moreover, the study on the strength of flocs might provide some knowledge in predicting the movement of colloidal particles. However, some limitations are worth remarking. Furthermore, in the real soil system, the interaction between particles might become more complex with the surrounding factors. In future works, more parameters should be considered to study the interaction between protein and humic acid as a complex polyelectrolyte-like particle.

## Supplementary Materials

The following are available online at https://www.mdpi.com/2073-4360/12/8/1770/s1, Figure S1: Surface charge density between lysozyme (0.5 mM and 0.1 M KCl) and leonardite humic acid (0.1 M NaCl) at the function of pH, Figure S2: Early aggregation stage after 2 h of addition of LSZ to LHA solution of the LSZ–LHA suspension at mass ratio CLSZ/CLHA = 2.5 at 3 mM (A), 10 mM (B), and 50 mM (C) KCl concentration, Figure S3: Microscopic view on the LSZ–LHA complex with mass ratio CLSZ/CLHA = 2.5 at 3 mM (A), 10 mM (B), and 50 mM (C) KCl concentration (the scale bars indicate 50 μm). The pH were at the charge neutralization region, Figure S4: Microscopic view on the LSZ–LHA complex with mass ratio CLSZ/CLHA = 5 at 10 mM KCl concentration (the scale bars indicate 50 μm), Table S1: Debye length of different KCl concentrations (3 mM, 10 mM, and 50 mM) use in the LSZ–LHA system.
