# Supplementary material for: Strength of Flocs Formed by the Complexation of Lysozyme with Leonardite Humic Acid
Source: Polymers (Basel). 2020 Aug 7;12(8):1770. doi: 10.3390/polym12081770 (PMC7463964; doi:10.3390/polym12081770)
Supplement: Supplementary file 1 [file polymers-12-01770-s001.pdf]

polymers

Supplementary Information

Strength Flocs Formed by the Complexation of Lysozyme with Leonardite Humic Acid

Wan Khairunnisa Wan Abdul Khodir [1], Azizul Hakim [2], and Motoyoshi Kobayashi [3]*

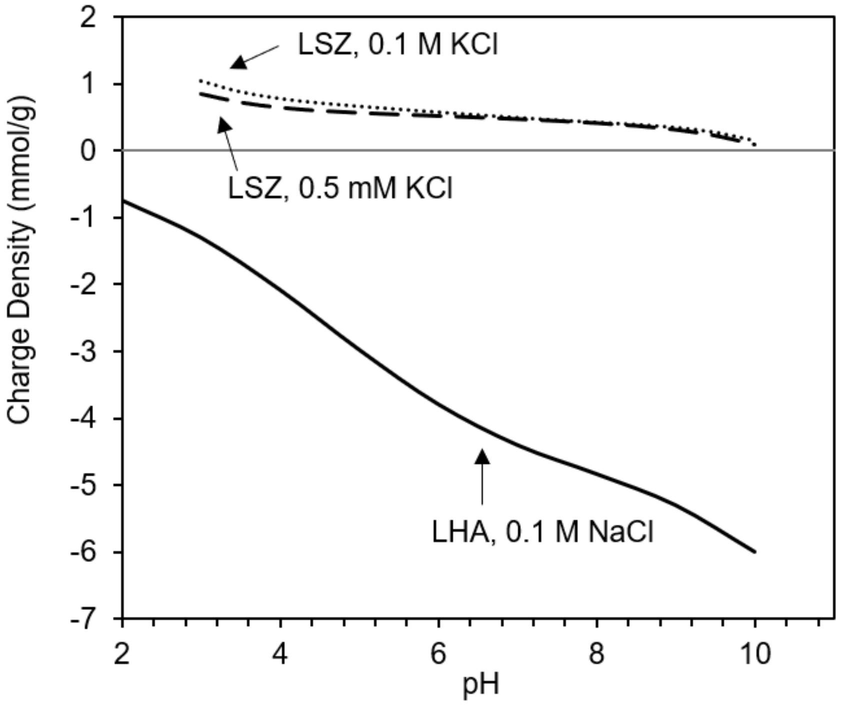

Figure S1. Surface charge density between lysozyme (0.5 mM and 0.1 M KCl) and Leornadite humic acid (0.1 M NaCl) at the function of pH [1–6].

Table S1. Debye length of different KCl concentrations (3 mM, 10 mM and 50 mM) use in the LSZ-LHA system.

Concentration of KCl (mM)	3	10	50
**Debye Length (nm)**	5.55	3.04	1.36

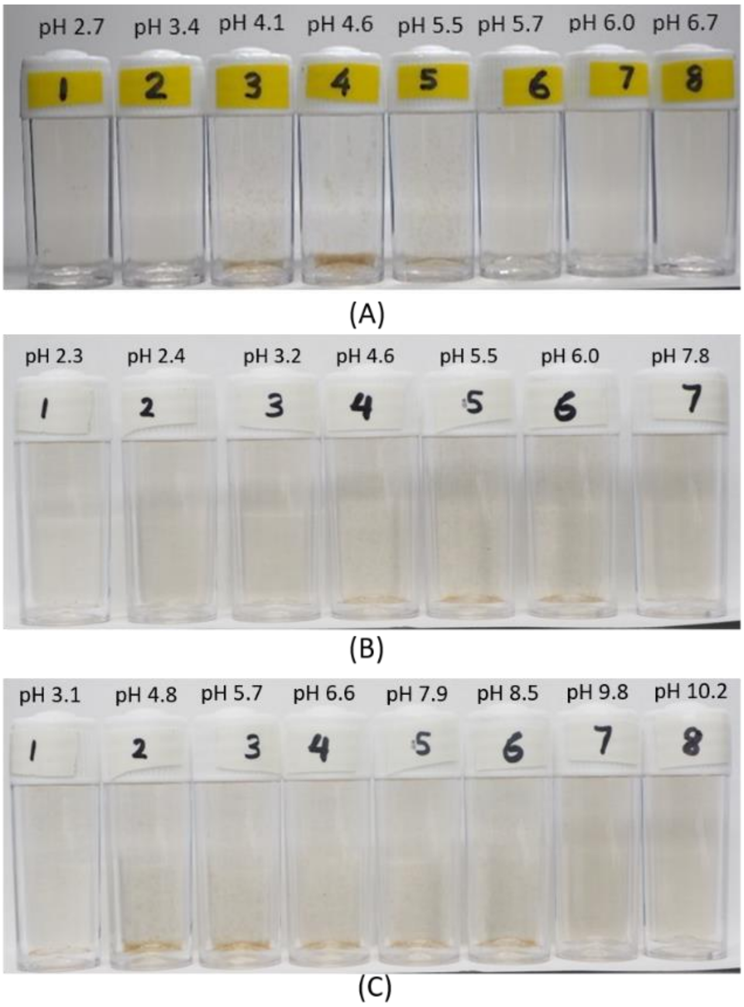

Figure S2. Early aggregation stage after 2 hours of addition of LSZ to LHA solution of LSZ-LHA suspension at mass ratio C_{LSZ}/C_{LHA} = 2.5 at 3 mM **(A)**, 10 mM **(B)** and 50 mM **(C)** KCl concentration.

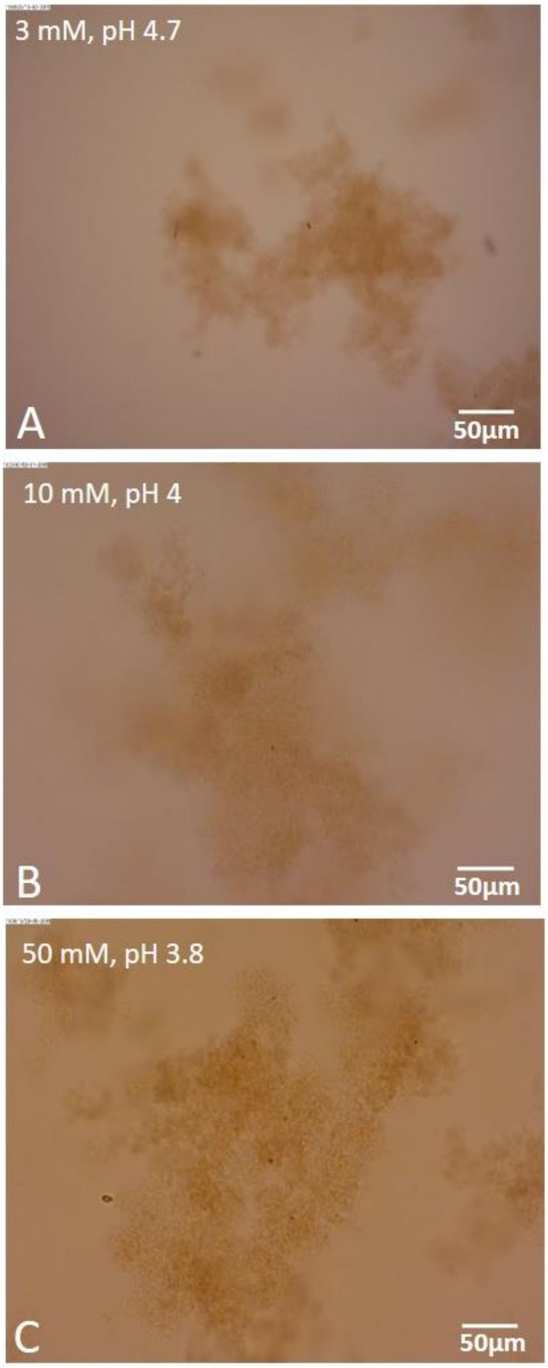

Figure S3. Microscopic view on LSZ-LHA complex with mass ratio C_{LSZ}/C_{LHA} = 2.5 at 3 mM **(A)**, 10 mM **(B)** and 50 mM **(C)** KCl concentration (the scale bars indicate 50 μm). The pHs were at the charge neutralization region.

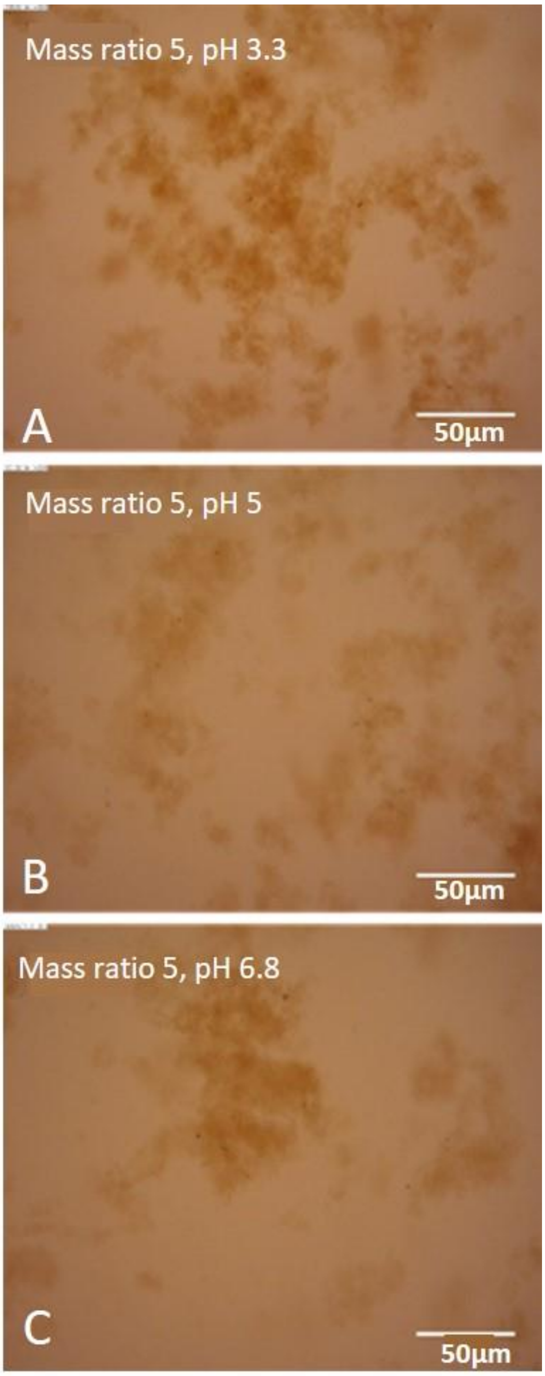

Figure S4. Microscopic view on LSZ-LHA complex with mass ratio C_{LSZ}/C_{LHA} = 5 at 10 mM KCl concentration (the scale bars indicate 50 μm).

References

1. Yamaguchi, A.; Kobayashi, M. Quantitative Evaluation of Shift of Slipping Plane and Counterion Binding to Lysozyme by Electrophoresis Method. *Colloid Polym. Sci.* **2016**, *294*, 1019–1026. https://doi.org/10.1007/s00396-016-3852-4.
2. Hakim, A.; Kobayashi, M. Aggregation and Charge Reversal of Humic Substances in the Presence of Hydrophobic Monovalent Counter-Ions: Effect of Hydrophobicity of Humic Substances. *Colloids Surfaces A Physicochem. Eng. Asp.* **2018**, *540*, 1–10. https://doi.org/10.1016/j.colsurfa.2017.12.065.
3. Li, Y.; Tan, W.; Koopal, L.K.; Wang, M.; Liu, F.; Norde, W. Influence of Soil Humic and Fulvic Acid on the Activity and Stability of Lysozyme and Urease. *Environ. Sci. Technol.* **2013**, *47*, 5050–5056. https://doi.org/10.1021/es3053027.

4. Kuehner, D.E.; Engmann, J.; Fergg, F.; Wernick, M.; Blanch, H.W.; Prausnitz, J. M. Lysozyme Net Charge and Ion Binding in Concentrated Aqueous Electrolyte Solutions. *J. Phys. Chem. B* **1999**, *103*, 1368–1374. https://doi.org/10.1021/jp983852i.
5. Norde, W.; Gonzalez, F.G.; Haynes, C.A. Protein Adsorption on Polystyrene Latex Particles. *Polym. Adv. Technol.* **1995**, *6*, 518–525. https://doi.org/10.1002/pat.1995.220060713.
6. Tan, W.F.; Koopal, L.K.; Weng, L.P.; van Riemsdijk, W.H.; Norde, W. Humic Acid Protein Complexation. *Geochim. Cosmochim. Acta* **2008**, *72*, 2090–2099. https://doi.org/10.1016/j.gca.2008.02.009.
